# Application of the Taguchi Method for Optimizing the Process Parameters of Producing Lightweight Aggregates by Incorporating Tile Grinding Sludge with Reservoir Sediments

**DOI:** 10.3390/ma10111294

**Published:** 2017-11-10

**Authors:** How-Ji Chen, Sheng-Nan Chang, Chao-Wei Tang

**Affiliations:** 1 Department of Civil Engineering, National Chung-Hsing University, No. 250, Kuo Kuang Road, Taichung 402, Taiwan; hojichen@nchu.edu.tw (H.-J.C.); alex.csn@gmail.com (S.-N.C.); 2 Department of Civil Engineering & Geomatics, Cheng Shiu University, No. 840, Chengcing Rd., Niaosong District, Kaohsiung 83347, Taiwan

**Keywords:** lightweight aggregate, reservoir sediments, grinding sludge, orthogonal array, Taguchi method

## Abstract

This study aimed to apply the Taguchi optimization technique to determine the process conditions for producing synthetic lightweight aggregate (LWA) by incorporating tile grinding sludge powder with reservoir sediments. An orthogonal array *L*_16_(4^5^) was adopted, which consisted of five controllable four-level factors (i.e., sludge content, preheat temperature, preheat time, sintering temperature, and sintering time). Moreover, the analysis of variance method was used to explore the effects of the experimental factors on the particle density, water absorption, bloating ratio, and loss on ignition of the produced LWA. Overall, the produced aggregates had particle densities ranging from 0.43 to 2.1 g/cm^3^ and water absorption ranging from 0.6% to 13.4%. These values are comparable to the requirements for ordinary and high-performance LWAs. The results indicated that it is considerably feasible to produce high-performance LWA by incorporating tile grinding sludge with reservoir sediments.

## 1. Introduction

Lightweight aggregate (LWA) is a general term for natural or artificial aggregates with a bulk density of less than 1200 kg/m^3^ [1]. LWA can be used to replace ordinary aggregate to produce lightweight aggregate concrete (LWAC), which has the advantages of being lightweight, heat insulating, and having strong fire and seismic resistance [2]. As a result, LWA has long been used by many countries as a building material [1,2]. Depending on the source, lightweight aggregate (LWA) can be divided into two categories: natural LWA and artificial LWA [1,2,3]. Natural LWA comes from naturally-formed porous rocks, such as pumice, volcanic slag, and vermiculite [1]. Artificial LWA is mainly produced by expanding raw materials such as shale, clay, slate, and phyllite under heat [1]. Current LWAs are sintered at high temperatures of around 1000–1200 °C. The use of a high-temperature process has the following advantages: high yield, low cost, stable quality, and high thermal efficiency. Furthermore, LWAs can also be obtained with alternative low-temperature processes, such as cold bonding pelletization [4,5,6,7,8]. The cold bonding method uses normal water curing at an ambient temperature to bond the mixing material. The bonding achieved from this method is more rigid and has higher drying shrinkage [8]. However, the disadvantages of this method can be overcome by using compaction during agglomeration techniques. Moreover, compared with sintered LWAs, cold-bonded LWAs are mainly used for structural insulated concrete, lightweight blocks, and wall panels.

Wall and floor tiles belong to the fine ceramics category. In the production process of polished homogeneous tiles, they are ground with an abrasive (consisting of magnesium and silicon carbide) to provide a smooth surface. During the grinding process, the surface of the polished tiles is peeled off due to the friction of the abrasive, and the abrasives are peeled off due to abrasion [9]. The resulting dust is taken to the wastewater treatment plant along with the rinse water, where it becomes a sludge cake by means of collection, concentration, and press filtration procedures. This type of solid waste generated during the production of polished homogeneous tiles is called grinding sludge, which are very fine powders. In light of the growing concern regarding the depletion of nonrenewable resources and waste management, grinding sludge should be effectively utilized. However, grinding sludge as a raw material of polished homogeneous tiles could lead to high-temperature expansion of the finished product and produce a large number of holes, as well as present other issues [9]. At present, grinding sludge is only used as a backfill material or abandoned in landfill. Therefore, there is an urgent need to develop novel reuse applications for grinding sludge in Taiwan.

For the ecological protection and efficient use of waste, the production of LWAs using incinerator bottom ash, fly ash, sewage sludge, reservoir sediment, coconut shells, water purification sludge, waste thin film transition liquid crystal displays, textile sludge, paper sludge, non-metallic automotive shredder residues, recycled polyolefin waste, cement kiln dust, etc., has attracted many scholars to engage in related research [9,10,11,12,13,14,15,16,17,18,19,20,21,22,23,24,25,26,27,28,29,30,31,32,33,34,35,36,37,38,39,40,41]. Hsu explored the purification and recycling of grinding sludge [9]. His experimental results indicated that vitrification in the recycling samples was significant and led to the formation of more micropores, which were in proportion to the added amount of grinding sludge and sintering temperatures. Farías et al. investigated the effects of sieved waste generated from the brewing industry on LWAs manufactured with clay [37]. Their tests showed that the resulting aggregate had low bulk density and increased water absorption and porosity. Colangelo et al. dealt with the design and characterization of concrete mixtures containing artificial aggregates obtained through the cold-bonding pelletization of the non-metallic fraction of automotive shredder residues, which was pelletized with blended cementitious mixtures containing coal fly ash [38]. Their experimental results showed good mechanical properties in concrete containing these aggregates. In other words, industrial waste or municipal solid waste can be reused as a sustainable resource in the manufacturing process of artificial LWA [32,34,37]. However, there are currently no publications on the topic of LWAs produced with grinding sludge and reservoir sediments.

Factors affecting the engineering properties of sintered LWAs include raw material composition and firing conditions [2,19,21,22,32]. However, as the design parameters or parameter levels increase, the time and cost of the experiment increase significantly and the experimental conditions become more complex. Instead of having to test all possible combinations, like factorial design, the Taguchi method only tests pairs of combinations [42,43,44,45]. This is mainly due to the Taguchi’s orthogonal arrays being highly fractional orthogonal designs [42]. Therefore, these designs can be used to estimate the main effects using only a minimum amount of experimentation, thus saving time and resources [42,43,44,45].

In view of the above considerations, the present study aimed at conducting an investigation on the development of LWAs by incorporating tile grinding sludge with reservoir sediments. The experiments were designed using an orthogonal array technique in an *L*_16_ array with five controllable four-level factors. Moreover, by studying the effect of individual factors on the results, the best factor combination was determined.

## 2. Experimental Details

### 2.1. Characterization of the Raw Materials

The main materials used included tile grinding sludge and reservoir sediments. The grinding sludge was collected from a polished homogeneous tile factory located in Central Taiwan, while the reservoir sediments were collected from the Shihmen Reservoir located in Northern Taiwan. To satisfy the requirements for pelletizing and sintering, the sludge was thoroughly milled prior to mixing with reservoir sediments. The characteristics of the raw materials were determined, including particle distribution [46], hydrometer analysis [47], specific gravity [48], chemical composition [49], and ignition loss. The characteristics of the aggregates were also determined including the particle density, water absorption [50], bloating index, and ignition loss.

### 2.2. Experimental Program

As previously mentioned, the Taguchi method can effectively reduce the number of tests required in a design procedure [42,43,44,45]. Therefore, the Taguchi method was adopted in this research to design the experimental protocol. In this study, sludge content, preheat temperature, preheat time, sintering temperature, and sintering time were selected as the process parameters. The interaction between the process parameters was neglected as the Taguchi method has the ability to detect the presence or absence of interaction [36,51,52]. The levels of these parameters are given in Table 1. In addition, particle density, water absorption, bloating ratio, and loss on ignition of the resulting aggregate were used as the performance parameters for this research.

An orthogonal array (OA), *L*_16_(4^5^), and five controllable four-level factors were adopted (Table 2). The effects of the composition and firing conditions on the properties of the resulting aggregate were evaluated with a laboratory-scale setup. Moreover, the range analysis and analysis of variance were the mathematical statistics used to explore the effects of the experimental factors on the performances of the produced LWAs, thus optimizing the selected parameters.

### 2.3. Preparation of Aggregate Pellets and Aggregate Sintering

Prior to pelletizing, samples of the raw materials were dried at room temperature. The samples were then crushed and milled using a leading-coming-air type crushing machine or a dry ball mill. The resulting fine powders were thoroughly mixed to ensure homogeneity as per the design ratio in Table 2. Subsequently, according to [19,23,32], an appropriate amount of water (20–25% by weight mixture) was added to the mixture, then approximately spherical particles with a diameter of 12–15 mm were produced using a disk pelletizer.

The formed pellets were dried in an oven at 105 °C for 24 h and then fired in an electric laboratory kiln. The firing of the synthetic aggregates included preheating and sintering. The main equipment used in this study was a self-designed electric laboratory kiln, which was static, had a programmable control, and two chambers. The dried pellets on an alumina base were placed in the preheating chamber of the kiln and heated to the target temperature for different dwell times. The preheated pellets were subsequently placed in the sintering chamber of the kiln and heated at the desired maximum temperature for different dwell times, then quenched in air.

### 2.4. Test Methods and Data Analysis

As previously described, the characteristics of the resulting aggregates were evaluated by four performance parameters (particle density, water absorption, swelling rate, and ignition loss). The particle density of the sintered aggregate pellets were determined by the Archimedes principle, as shown below [36,53]:

(1) $$\rho_{p}=\frac{m_{dry}}{m_{sat}-m_{imm}}$$

where *ρ_p_* = particle density (g/cm^3^); *m_dry_* = dry mass (g/cm^3^); *m_imm_* = immersed mass (g/cm^3^); and *m_sat_* = 24-h saturated surface-dry mass (g/cm^3^).

The water absorption was determined by dipping the sintered aggregate pellets in water for 24 h and calculated from the following formula [50]:

(2) $$\mathrm{Water}\;\mathrm{absorption}\;\left(W_{a}\right)=\frac{m_{sat}-m_{dry}}{m_{dry}}\times 100\%$$

To explore the volume change of the pellets, the particle size of the pellets before and after sintering was measured. The bloating ratio was defined as the ratio of the volume of the fired pellet to the volume of the unfired pellet, which was calculated as follows [36,53]:

(3) $$\mathrm{Bloating}\;\mathrm{ratio}\;\left(B_{r}\right)=\frac{V_{a}}{V_{p}}\times 100\%$$

where *V_p_* = initial volume of the green pellet; and *V_a_* = volume of the sintered aggregate pellet.

The loss on ignition was defined as the mass loss of the dried pellets after firing and was expressed as a percentage of the total initial mass.

To evaluate the effect of each selected factor on the quality characteristics being studied, it was necessary to calculate the signal-to-noise ratio (*S*/*N* ratio) for each control factor [42]. The *S*/*N* ratio is calculated by the mean squared deviation (*MSD*) [53]. In this study, the observed values of particle density, water absorption, and loss of ignition were set to a minimum level, while the observed values of the bloating ratio were set to a maximum level. In other words, the *S*/*N* ratio was chosen according to the-smaller-the-better criterion or the-larger-the-better quality characteristics. Basically, the aim of any experiment is to determine the highest possible *S*/*N* ratio for the result. If the *S*/*N* ratio (*η*) for the-smaller-the-better target for all the responses is expressed in decibels (dB), it can be defined by a logarithm based on the *MSD* around the target value, as shown below [36,53]:

(4) $$\eta =-10\times \log_{10}\left(MSD\right)=-10\times \log_{10}\left(\sum_{i=1}^{n}y_{i}^{2}/n\right)$$

where *n* is the number of repetitions or observations; and *y_i_* is the observed data. In the case of the-larger-the-better target, the *S*/*N* ratio (*η*) is generally derived from the reciprocal of its quality characteristics value, as shown below [36,53]:

(5) $$\eta =-10\times \log_{10}\left(MSD\right)=-10\times \log_{10}\left(\sum_{i=1}^{n}1/y_{i}^{2}/n\right)$$

In addition, the analysis of variance, popularly known as the ANOVA, was used to detect the optimization of the observed values. The total sum of the squared deviations (*SS_T_*) from the *S/N* ratio can be calculated from the following equation [36,42,43,44,45]:

(6) $$SS_{T}=\sum_{i=1}^{n}{\left(\eta_{i}-\eta_{m}\right)}^{2}$$

where *n* is the number of experiments in the orthogonal array; *η_i_* is the mean *S/N* ratio for the *i*th experiment; and *η_m_* is the grand mean of the *S/N* ratio. The sum of the squares from the tested parameter *Z* (*SS_Z_*) can be calculated from the following equation [36,42,45]:

(7) $$SS_{Z}=\sum_{j=1}^{r}\frac{Z_{j}^{2}}{t}-\frac{1}{n}{\left[\sum_{i=1}^{n}\eta_{i}\right]}^{2}$$

where *Z* is one of the tested parameters; *j* is the level number of parameter *Z*; *r* is the number of levels of parameter *Z*; *t* is the repeating number of each level of parameter *Z*; and *Z_j_* is the sum of the *S*/*N* ratio involving parameter *Z* and level *j*. As for the sum of the squares from the error parameters (*SS_e_*), it can be calculated from the following equation [36,42,43,44,45]:

(8) $$SS_{e}=SS_{T}-SS_{F}$$

where *SS_F_* is the sum of squared deviations due to each parameter.

Moreover, a statistical *F* test was used to determine which process parameters had a significant effect on the performance characteristic. To perform the *F* test, the mean of square deviation (variance) due to each process parameter and error term needed to be calculated as follows [36,42,43,44,45]:

(9) $$MS_{Z}=SS_{Z}/df_{Z}$$

(10) $$MS_{e}=SS_{e}/df_{e}$$

where *MS_Z_* is the mean of square deviation due to parameter *Z*; *df_Z_* is the degree of freedom of parameter *Z*; *MS_e_* is the mean squared deviation due to error term; and *df_e_* is the degree of freedom of the error term; then, the *F* ratio of parameter *Z* (*F_Z_*) is calculated from the following equation [36,42,43,44,45]:

(11) $$F_{Z}=MS_{Z}/MS_{e}$$

The corrected sum of the squares (SS_Z_^*^) can be calculated as:

(12) $$SS_{Z}{}^{*}=SS_{Z}-MS_{e}\times df_{Z}$$

As for the percentage contribution of parameter *Z* (*P_Z_*), it can be calculated as:

(13) $$P_{Z}=SS_{Z}{}^{*}/SS_{T}$$

## 3. Results and Discussion

### 3.1. Raw Materials

**Figure 1:**
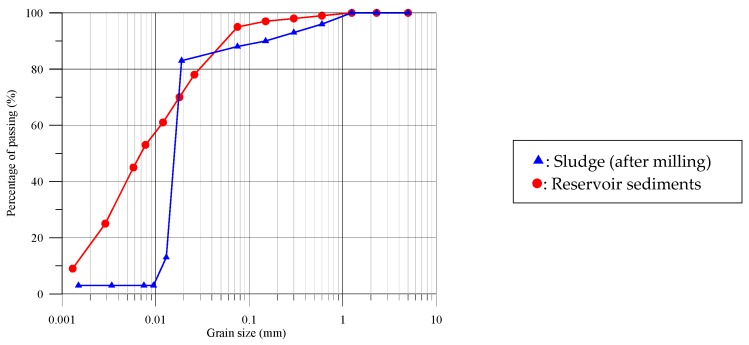
Grain size distributions of sludge and reservoir sediments.

The particle size distribution characteristics for the tile grinding sludge and reservoir sediments with particles less than 75 μm in size have a close relationship with the driving force required to reach the sintered state [19]. Therefore, a hydrometer analysis was conducted [47]. Subsequently, the complete particle size distributions of the sludge and reservoir sediments were plotted by combining the results of sieve analysis and hydrometer analysis, as shown in Figure 1. It can be observed from Figure 1 that the D_50_ particle sizes for the reservoir sediments was 0.007 mm, which is the median diameter or the medium value of the particle size distribution, while the sludge had a larger portion of coarse particles.

Table 3 shows that the specific gravity for the sludge and reservoir sediments was 2.02 and 2.75, respectively. In addition, the plasticity index (PI) for the sludge and reservoir sediments was 7 and 10, respectively. These indicated that the sediments had proper plasticity, while the plasticity of the sludge was slightly worse. On the whole, the higher the PI, the greater the plasticity of the material, making the pelletizing process easier [19]. Therefore, a large proportion of the designed mixture was comprised of the reservoir sediment for this research, while the sludge was used as an additive and was incorporated into the reservoir sediments to produce LWAs.

**Figure 2:**
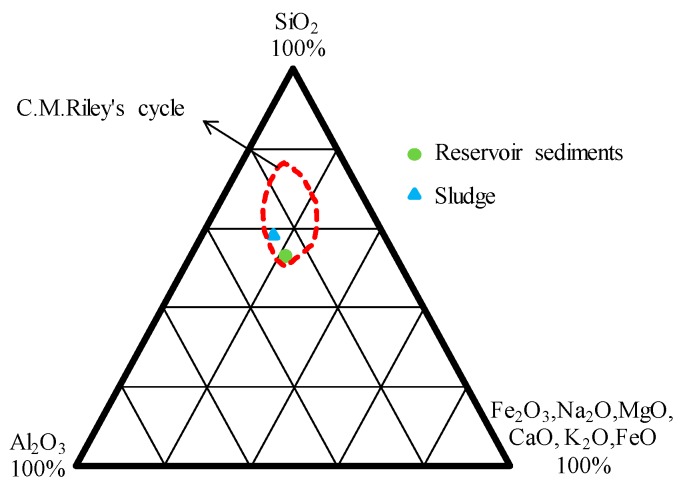
Ternary diagram of sludge and reservoir sediments.

In general, artificial LWAs are formed by heating an appropriate material rapidly so that it bloats when reaching sintering temperatures. A suitable expansion material must meet two requirements [54]: it must contain substances that develop gases at high temperature, and produce a highly viscous liquid phase at high temperature that can entrap the gases. Table 4 shows the chemical composition of the raw materials used. The sludge had a high SiO_2_ content (64.3%) and low concentrations of fluxing (Fe_2_O_3_ + CaO + MgO + K_2_O + Na_2_O, 12.4%), while the reservoir sediments showed a lower SiO_2_ content (53.4%) and high values of fluxing (21.8%). Basically, SiO_2_ and Al_2_O_3_ ensures a high viscosity liquid phase at high temperatures, which can entrap the gas. In contrast, the fluxing influences the softening and melting temperatures of aggregates [54]. On the whole, the analysis results fell in the area of the expandable region of the ternary (SiO_2_/Al_2_O_3_/fluxing) diagram produced by Riley (Figure 2). This indicated that the sludge and sediments used were feasible for sintering LWAs.

### 3.2. Particle Density

Table 5 shows the experimental results and the corresponding *S*/*N* ratios using Equation (4) or (5). As the experimental design was orthogonal, the effect of each parameter used could be separated at different levels. Taking the sludge content as an example, the mean *S*/*N* ratio at levels 1, 2, 3, and 4 were calculated by averaging the *S*/*N* ratios of experiments 1–4, 5–8, 9–12, and 13–16, respectively. As for the other parameters, the mean *S*/*N* ratio at each level was computed in a similar manner. The influence of each selected factor on the quality characteristic investigated is described in detail below.

**Figure 3:**
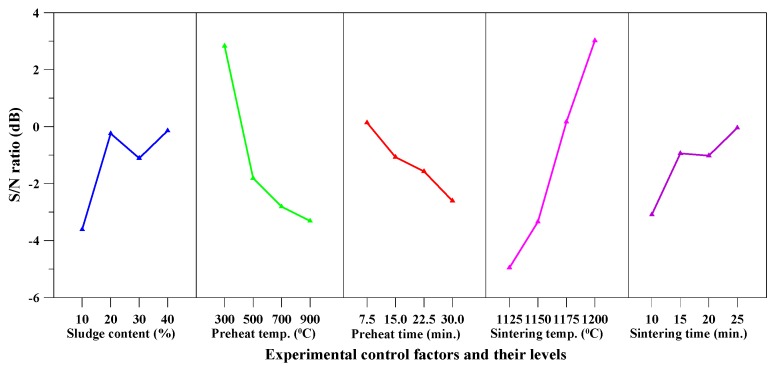
*S*/*N* response graph for particle density.

Table 5 shows that the particle density of the produced aggregate ranged between 0.43 and 2.1 g/cm^3^. Moreover, the lowest value of particle density was around 0.43 g/cm^3^ (experiment number F9). Table 6 (i.e., the response table) shows the mean *S*/*N* ratio at each level of the parameters (A–E) for particle density, while Figure 3 shows the *S*/*N* response graph for particle density. As shown in Equation (4), the larger the *S*/*N* ratio, the smaller the variance of particle density around the desired (the-smaller-the-better) value. From Table 6 and Figure 3, it can be seen that the sintering temperature was the most important factor affecting the responses; the maximum value of response was at the highest level of the sintering temperature.

The results of the ANOVA of particle density are given in Table 7. In addition, the *F* values were obtained for a 95% level of confidence and the percentage contribution of each parameter was also calculated. The sintering temperature was the most significant factor that contributed to the total particle density of the aggregate. The main contributions from the parameters were: sintering temperature (43.78%), preheat temperature (25.1%), and preheat time (24.7%). Thus, based on the results of the *S*/*N* ratio and ANOVA analyses, the optimal combination of parameters and their levels for achieving minimum particle density was A_4_B_1_C_1_D_4_E_4_, i.e., sludge content at level 4, preheat temperature at level 1, preheat time at level 1, sintering temperature at level 4, and sintering time at level 4.

### 3.3. Water Absorption

**Figure 4:**
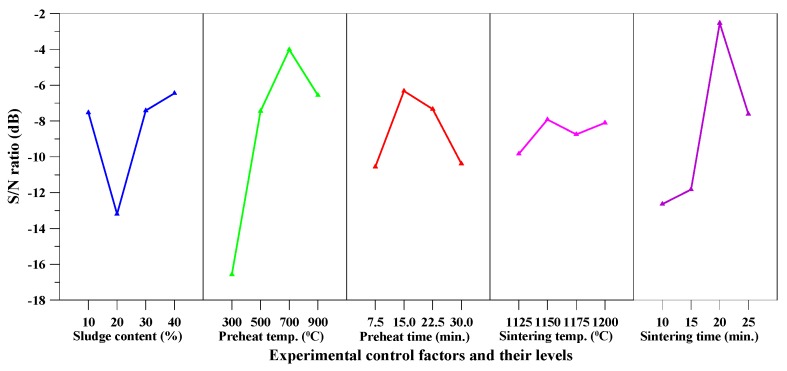
*S*/*N* response graph for water absorption.

It can be seen from Table 5 that the water absorption of the produced aggregate ranged between 0.6–13.4%. Moreover, some aggregates were impervious to water (water absorption below 1%). In particular, the lowest value of water absorption was around 0.6% and was obtained with Sample F3. Table 8 shows the mean *S*/*N* ratio at each level of the parameters for water absorption, while Figure 4 shows the *S*/*N* response graph for water absorption. It is evident from Table 8 and Figure 4 that the preheating temperature was the most critical factor affecting water absorption; the maximum value of response was at the highest level of sludge content. Figure 4 also indicated that the water absorption decreased with an increase of sintering time.

The results of the ANOVA of water absorption are given in Table 9. As can be easily seen from Table 9, the preheating temperature was the most significant factor that contributed to the total water absorption of the aggregate. The contributions from these parameters were: preheat temperature (44.12%), sintering time (31.33%), sludge content (13.09%), and preheat time (11.46%). Therefore, based on the results of the *S*/*N* ratio and ANOVA analyses, the optimal combination of parameters and their levels for achieving minimum water absorption was A_4_B_3_C_2_D_2_E_3_, namely sludge content at level 4, preheat temperature at level 3, preheat time at level 2, sintering temperature at level 2, and sintering time at level 3.

### 3.4. Bloating Ratio

**Figure 5:**
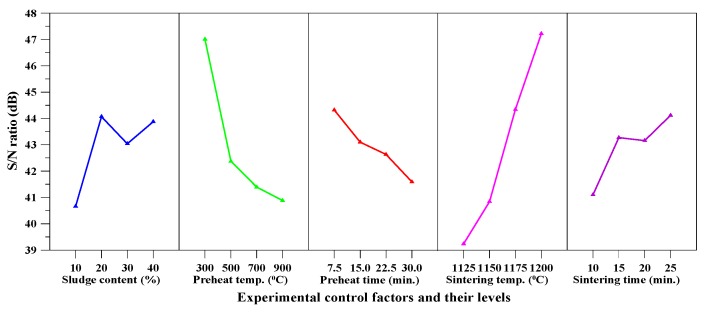
*S*/*N* response graph for bloating ratio.

Table 5 shows that the bloating ratio of the produced aggregate ranged between 77.4% and 377.4%. In other words, the largest value of bloating ratio was around 377.4% and was obtained with Sample F9. Table 10 shows the mean *S*/*N* ratio at each level of the parameters for bloating ratio, while Figure 5 shows the *S*/*N* response graph for bloating ratio. From Table 10 and Figure 5, it can be seen that the sintering temperature was the most important factor affecting bloatability; the maximum value of response was at the highest level of sintering temperature. In addition, Figure 5 also indicated that the bloating ratio increased with the rise in sintering temperature.

The results of the ANOVA of bloating ratio are given in Table 11. The contributions from these parameters were: sintering temperature (44.23%), preheat temperature (25.19%), preheat time (24.73%), and sludge content (4.5%). As a result, according to the results of the *S*/*N* ratio and ANOVA analyses, the optimal combination of parameters and their levels for achieving the maximum bloating ratio was A_2_B_1_C_1_D_4_E_4_, i.e., sludge content at level 2, preheat temperature at level 1, preheat time at level 1, sintering temperature at level 4, and sintering time at level 4.

### 3.5. Loss on Ignition

**Figure 6:**
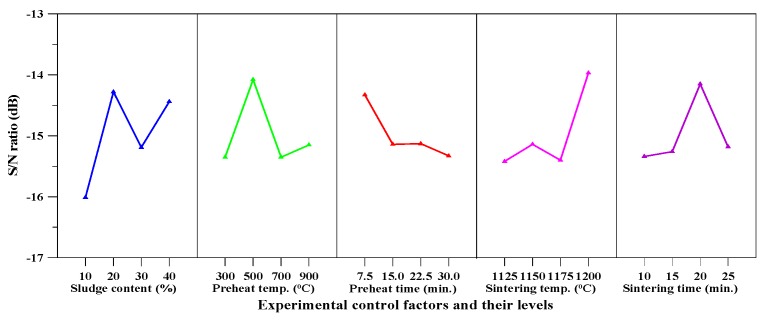
*S*/*N* response graph for loss on ignition.

From Table 5, it is clear that the loss of ignition of the produced aggregate ranged between 3.5% and 6.7%. Moreover, the lowest value of loss on ignition was around 3.5% and was obtained with Sample F6. Table 12 shows the mean *S*/*N* ratio for each level of the parameters for loss on ignition, while Figure 6 shows the *S*/*N* response graph for loss of ignition. From Table 12, it can be seen that the sludge content was the most significant factor in controlling ignition loss.

The results of the ANOVA of loss on ignition are given in Table 13. The contributions from these parameters were preheat time (49.84%), sludge content (21.84%), sintering temperature (13.89%), and preheat temperature (8.70%). Accordingly, based on the results of the *S*/*N* ratio and ANOVA analyses, the optimal combination of parameters and their levels for achieving minimum loss on ignition is A_2_B_2_C_1_D_4_E_3_, that is, sludge content at level 2, preheat temperature at level 2, preheat time at level 1, sintering temperature at level 4, and sintering time at level 3.

### 3.6. Discussion

**Figure 7:**
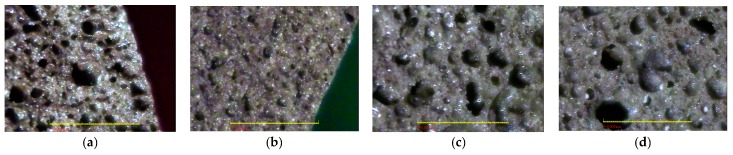
The internal cellular pore system of the sintered LWA. (**a**) experiment number F4; (**b**) experiment number F11; (**c**) experiment number F15; and (**d**) experiment number F16.

The aggregate density of the fired aggregates in this study ranged from 0.43 to 2.1 g/cm^3^ and their water absorption ranged from 0.6% to 13.4%. The particle density and water absorption of the three main synthetic LWAs are shown in Table 14 [1,2,3,19,21,22]. From this point of view, the fired aggregates have been consistent with the basic requirements of LWA. Moreover, according to Figure 3, Figure 4, Figure 5 and Figure 6, the target properties and corresponding optimal process are tabulated in Table 15. On the other hand, to meet the characteristics (lightweight and low water absorption) of high-performance LWAs, the experimental results of the experimental combinations F4, F11, F15, and F16 were further analyzed. Typical results of the optical microscopy image analysis of these experimental combinations are shown in Figure 7. The micrographs show a well-formed, dense matrix material that contains a significant volume of isolated approximately spherical porosity. In addition, the particle density and water absorption of the experimental combinations F4, F11, F15, and F16 were compared with those of commercially-available LWAs, i.e., Norwegian Leca™ (0–4 mm, Saint-Gobain, Oslo, Norway) and Liapor™ 8 (4–8 mm, Liapor, Pautzfeld, Germany). Table 16 shows that the particle density of F4, F15, and F16 was 0.89–1.27 g/cm^3^ and was lower than that of Leca™ and Liapor™ 8. Moreover, it can be clearly seen that the water absorption of F4, F11, F15, and F16 was 1.4–3.0% and was significantly lower than that of Leca™ and Liapor™ 8. This was in agreement with the uniform pore structure with small isolated voids in the aggregates. As a result, it was concluded that the experimental combinations F4, F11, F15, and F16 were suitable for use as high-performance LWAs, which had low particle density and water absorption due to a uniformly-distributed system of pores.

The laboratory-scale test results showed that the use of tile grinding sludge with reservoir sediments could produce high-performance LWAs. According to the process parameters and design levels in Table 15, the aggregate with the smallest particle density, water absorption, and loss on ignition could be produced separately. In addition, the aggregate with the largest bloating ratio could also be produced. However, from the test results in Table 5, it can be seen that the aggregate with a low particle density and a high bloating ratio had a higher water absorption. These aggregates were more suitable as thermal insulation materials. By contrast, the aggregate with a low bloating ratio showed a relatively larger particle density, which was unable to meet the lightweight requirements. Therefore, this study selected an experimental combination to explore the feasibility of producing high-performance LWAs by using commercial tunnel kiln equipment. The brick-fired tunnel kiln consisted of three parts: preheating, firing, and cooling; and was a continuous moving ware kiln where the clay products to be fired were passed on cars through a long horizontal tunnel. To meet the characteristics of lightweight and low water absorption, an experimental combination with a lower particle density and a lower water absorption was selected from Table 2. At this point, the F15 experimental combination was selected for mass production testing. In other words, sludge content was 40%, preheat temperature was 700 °C, preheat time was 15 min, sintering temperature was 1200 °C, and sintering time was 10 min.

**Figure 8:**
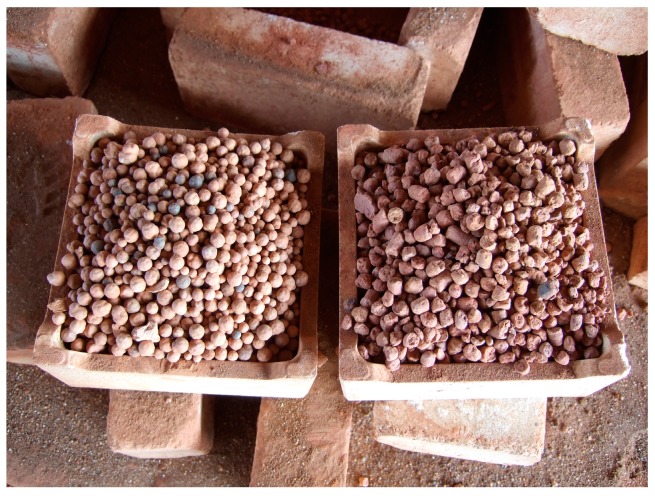
The sintered LWAs using the commercial tunnel kiln.

Table 17 shows the test results for high performance LWAs produced by the tunnel kiln. It can be seen that the properties of the high-performance LWAs met the requirements of general commercial specifications. The particle density of the synthetic aggregates (Figure 8) was 1.56 g/cm^3^, which was significantly lower than the normal density aggregate, and the 24-h water absorption was 1.4%. Moreover, its dry loose bulk density was 819 kg/m^3^, which met the requirements of ASTM C 330 [55] with a bulk density less than 880 kg/m^3^ for coarse aggregate. On the other hand, the synthetic aggregates were tested for crushing strength in accordance with GB/T 2842 [56]. The crushing strength of the synthetic aggregates was 12.4 MPa, which can be used to manufacture structural lightweight concrete, which for different types of applications such as high-rise buildings, concrete masonry, precast bridge deck, precast and prestressed concrete elements, etc.

## 4. Conclusions

This study presented an application of the Taguchi optimization technique in determining the process condition for producing synthetic LWAs by incorporating tile grinding sludge powder with reservoir sediments. Based on the above results and discussion, the following conclusions were drawn:

1. The aggregates manufactured in a laboratory had particle densities ranging from 0.46 to 2.10 g/cm^3^ and water absorption ranging from 0.6% to 13.4%. These values were comparable to the requirements for LWAs.
2. The analysis of variance method determined the impact of the experimental factors on the performance parameters and determined the optimum levels of each of the experimental factors.
3. The experimental combinations F4, F11, F15, and F16 were suitable for use as high-performance LWAs. The particle density of F4, F15, and F16 ranged from 0.89 to 1.27 g/cm^3^ and was lower than that of Leca™ and Liapor™ 8. Moreover, the water absorption of F4, F11, F15, and F16 ranged from 1.4% to 3.0% and was significantly lower than that of Leca™ and Liapor™ 8.
4. The particle density of the synthetic aggregates using a tunnel kiln was 1.56 g/cm^3^, which was significantly lower than the normal density aggregate. Moreover, its dry loose bulk density as 819 kg/m^3^, which meets the requirements of ASTM C 330 with bulk density less than 880 kg/m^3^ for coarse aggregate.
5. The experimental results indicate that it is possible to produce high performance LWAs by incorporating grinding sludge with reservoir sediments. Especially, the Taguchi method provides a simple, systematic, and efficient methodology for optimizing process conditions of synthetic LWAs by using grinding sludge and reservoir sediments and it drastically reduces the number of tests.

## Figures and Tables

**Table 1:** Process parameters and design levels.

Parameter (Experimental Control Factor)	Levels of Parameter				Performance Parameter
	1	2	3	4	
Sludge content, A (%)	10	20	30	40	Particle density (g/cm^3^) Water absorption (%) Bloating ratio (%) Loss on ignition (%)
Preheat temperature, B (°C)	300	500	700	900	
Preheat time, C (min)	7.5	15	22.5	30	
Sintering temperature, D (°C)	1125	1150	1750	1200	
Sintering time, E (min)	10	15	20	25	

**Table 2:** Orthogonal array for *L*_16_(4^5^).

Experiment Number	Parameter (Level)				
	A	B	C	D	E
F1	10 (1)	300 (1)	7.5 (1)	1125 (1)	10 (1)
F2	10 (1)	500 (2)	15 (2)	1150 (2)	15 (2)
F3	10 (1)	700 (3)	22.5 (3)	1175 (3)	20 (3)
F4	10 (1)	900 (4)	30 (4)	1200 (4)	25 (4)
F5	20 (2)	300 (1)	15 (2)	1175 (3)	25 (4)
F6	20 (2)	500 (2)	7.5 (1)	1200 (4)	20 (3)
F7	20 (2)	700 (3)	30 (4)	1125 (1)	15 (2)
F8	20 (2)	900 (4)	22.5 (3)	1150 (2)	10 (1)
F9	30 (3)	300 (1)	22.5 (3)	1200 (4)	15 (2)
F10	30 (3)	500 (2)	30 (4)	1175 (3)	10 (1)
F11	30 (3)	700 (3)	7.5 (1)	1150 (2)	25 (4)
F12	30 (3)	900 (4)	15 (2)	1125 (1)	20 (3)
F13	40 (4)	300 (1)	30 (4)	1150 (2)	20 (3)
F14	40 (4)	500 (2)	22.5 (3)	1125 (1)	25 (4)
F15	40 (4)	700 (3)	15 (2)	1200 (4)	10 (1)
F16	40 (4)	900 (4)	7.5 (1)	1175 (3)	15 (2)

**Table 3:** Physical test results of sludge and reservoir sediments.

Sample	Specific Gravity	Liquid Limit (%)	Plastic limit (%)	PI	Ingredients (wt %)			
					Gravels (>4.75 mm)	Sands (4.75–0.075 mm)	Silts (0.075–0.005 mm)	Clays (<0.005 mm)
Grinding sludge	2.02	26	19	7	0	12	84	4
Sediments	2.75	30	20	10	0	4	54	42

**Table 4:** Chemical composition of sludge and reservoir sediments.

Sample	Chemical Compositions (wt %)									
	SiO_2_	Al_2_O_3_	Fe_2_O_3_	CaO	MgO	K_2_O	Na_2_O	OS	LOI	Total
Sludge	64.3	21.1	1.0	1.3	4.8	3.8	2.7	-	2.8	100
Sediments	53.4	23.8	10.9	1.8	2.5	5.1	1.5	1.0	2.9	100

Notes: LOI = loss on ignition; OS = organic substance content.

**Table 5:** Experimental results and *S*/*N* ratio.

Experiment Number	Experimental Results				*S*/*N* Ratio (dB)			
	*ρ_p_* (g/cm^3^)	*W_a_* (%)	*B_r_* (%)	LOI (%)	*ρ_p_*	*W_a_*	*B_r_*	LOI
F1	1.51	13.4	108.2	6.7	−3.58	−22.54	40.68	−16.52
F2	1.92	2.1	85.1	6.1	−5.67	−6.44	38.6	−15.71
F3	1.54	0.6	106.3	6.4	−3.75	4.44	40.53	−16.12
F4	1.18	1.9	138.3	6.1	−1.44	−5.58	42.82	−15.71
F5	0.46	7.8	354.3	5.9	6.74	−17.84	50.99	−15.42
F6	0.55	2.3	298.3	3.5	5.19	−7.23	49.49	−10.88
F7	2.1	5.4	78.6	6.1	−6.44	−14.65	37.91	−15.71
F8	2.1	4.5	78.3	5.7	−6.44	−13.06	37.88	−15.12
F9	0.43	6.8	377.4	5.6	7.33	−16.65	51.54	−14.96
F10	1.47	4.0	109.6	5.9	−3.35	−12.04	40.8	−15.42
F11	1.27	1.4	126.9	5.8	−2.08	−2.92	42.07	−15.27
F12	2.08	0.8	77.4	5.7	−6.36	1.94	37.77	−15.12
F13	0.91	2.9	174.4	5.3	0.82	−9.25	44.83	−14.49
F14	1.48	1.6	107.1	5.2	−3.41	−4.08	40.6	−14.32
F15	0.89	1.4	178.9	5.2	1.01	−2.92	45.05	−14.32
F16	0.89	3.0	178.9	5.4	1.01	−9.54	45.05	−14.65

Note: *ρ_p_* = particle density; *W_a_* = water absorption; *B_r_* = bloating ratio; and LOI = loss on ignition.

**Table 6:** *S*/*N* response table for particle density.

Parameter	Mean *S*/*N* Ratio (*η*, Unit: dB)				Delta (Max. *η* − Min. *η*)	Rank
	Level 1	Level 2	Level 3	Level 4		
Sludge content, A (%)	−3.61	−0.24	−1.11	−0.14	3.47	3
Preheat temperature, B (°C)	2.83	−1.81	−2.81	−3.31	6.14	2
Preheat time, C (min)	0.14	−1.07	−1.57	−2.60	2.74	5
Sintering temperature, D (°C)	−4.95	−3.34	0.17	3.02	7.97	1
Sintering time, E (min)	−3.09	−0.94	−1.02	−0.04	3.05	4

**Table 7:** Analysis of variance and *F* test for particle density.

Parameter	Sum of Square (*SS_Z_*)	Degree of Freedom	Variance (*MS_Z_*)	*F* Value (*F_Z_*)	*F*_0.05;3,3_	Percentage Contribution (*P_Z_*)	Note
Sludge content, A (%)	31.34	3	10.45	2.02	9.28	5.02	
Preheat temperature, B (°C)	94.50	3	31.50	6.08	9.28	25.10	Significant
Preheat time, C (min)	15.54	3	5.18	1.00	9.28	24.70	Significant
Sintering temperature, D (°C)	153.28	3	51.09	9.86	9.28	43.78	Significant
Sintering time, E (min)	19.93	3	6.64	1.28	9.28	1.40	
All other/Error	15.54	3	5.18				
Total	314.59	15	104.86			100	

**Table 8:** *S*/*N* response table for water absorption.

Parameter	Mean *S*/*N* Ratio (*η*, Unit: dB)				Delta (Max. *η* − Min. *η*)	Rank
	Level 1	Level 2	Level 3	Level 4		
Sludge content, A (%)	−7.53	−13.2	−7.42	−6.45	6.75	3
Preheat temperature, B (°C)	−16.57	−7.45	−4.01	−6.56	12.56	1
Preheat time, C (min)	−10.56	−6.32	−7.34	−10.38	4.24	4
Sintering temperature, D (°C)	−9.83	−7.92	−8.75	−8.10	1.91	5
Sintering time, E (min)	−12.64	−11.82	−2.53	−7.61	10.11	2

**Table 9:** Analysis of variance and *F* test for water absorption.

Parameter	Sum of Square (*SS_Z_*)	Degree of Freedom	Variance (*MS_Z_*)	*F* Value (*F_Z_*)	*F*_0.05;3,3_	Percentage Contribution (*P_Z_*)	Note
Sludge content, A (%)	113.16	3	37.72	12.57	9.28	13.09	Sub significant
Preheat temperature, B (°C)	360.12	3	120.04	40.00	9.28	44.12	Significant
Preheat time, C (min)	55.17	3	18.39	6.13	9.28	11.46	Sub significant
Sintering temperature, D (°C)	9.00	3	3.00	1.00	9.28	0.00	
Sintering time, E (min)	258.32	3	86.11	28.69	9.28	31.33	Significant
All other/Error	9.00	3	3.00				
Total	795.77	15	265.26			100.00	

**Table 10:** *S*/*N* response table for bloating ratio.

Parameter	Mean *S*/*N* Ratio (*η*, Unit: dB)				Delta (Max. *η* – Min. *η*)	Rank
	Level 1	Level 2	Level 3	Level 4		
Sludge content, A (%)	40.66	44.07	43.04	43.88	3.41	3
Preheat temperature, B (°C)	47.01	42.37	41.39	40.88	6.13	2
Preheat time, C (min)	44.32	43.10	42.63	41.59	2.73	5
Sintering temperature, D (°C)	39.24	40.84	44.34	47.22	7.98	1
Sintering time, E (min)	41.10	43.27	43.16	44.12	3.02	4

**Table 11:** Analysis of variance and *F* test for bloating ratio.

Parameter	Sum of Square (*SS_Z_*)	Degree of Freedom	Variance (*MS_Z_*)	*F* Value (*F_Z_*)	*F*_0.05;3,3_	Percentage Contribution (*P_Z_*)	Note
Sludge content, A (%)	29.50	3	9.83	1.91	9.28	4.50	Sub significant
Preheat temperature, B (°C)	94.12	3	31.37	6.09	9.28	25.19	Significant
Preheat time, C (min)	15.45	3	5.15	1.00	9.28	24.73	Significant
Sintering temperature, D (°C)	153.59	3	51.20	9.94	9.28	44.23	Significant
Sintering time, E (min)	19.68	3	6.56	1.27	9.28	1.35	
All other/Error	15.45	3	5.15				
Total	312.33	15	104.11			100.00	

**Table 12:** *S*/*N* response table for loss on ignition.

Parameter	Mean *S*/*N* Ratio (*η*, Unit: dB)				Delta (Max. *η* – Min. *η*)	Rank
	Level 1	Level 2	Level 3	Level 4		
Sludge content, A (%)	−16.01	−14.28	−15.19	−14.44	1.73	1
Preheat temperature, B (°C)	−15.35	−14.08	−15.35	−15.15	1.27	3
Preheat time, C (min)	−14.33	−15.14	−15.13	−15.33	1.00	5
Sintering temperature, D (°C)	−15.42	−15.14	−15.40	−13.97	1.45	2
Sintering time, E (min)	−15.34	−15.26	−14.15	−15.18	1.19	4

**Table 13:** Analysis of variance and *F* test for loss on ignition.

Parameter	Sum of Square (*SS_Z_*)	Degree of Freedom	Variance (*MS_Z_*)	*F* Value (*F_Z_*)	*F*_0.05;3,3_	Percentage Contribution (*P_Z_*)	Note
Sludge content, A (%)	7.57	3	2.52	3.19	9.28	21.84	Significant
Preheat temperature, B (°C)	4.44	3	1.48	1.87	9.28	8.70	Sub significant
Preheat time, C (min)	2.37	3	0.79	1.00	9.28	49.84	Significant
Sintering temperature, D (°C)	5.68	3	1.89	2.39	9.28	13.89	Sub significant
Sintering time, E (min)	3.73	3	1.24	1.57	9.28	5.72	
All other/Error	2.37	3	0.79				
Total	23.80	15	7.93			100.00	

**Table 14:** Particle density and water absorption of three main synthetic LWAs [2].

Lightweight Aggregate Types	Particle Density (g/cm^3^)	Water Absorption (%)
Expanded clay	0.6–1.6	5–30
Expanded shale	0.4–1.2	5–15
Expanded fly ash	0.8–1.4	10–20

**Table 15:** Process parameters and design levels.

Performance Parameter	Experimental Control Factor				
	Sludge Content (%)	Preheat Temperature (°C)	Preheat Time (min)	Sintering Temperature (°C)	Sintering Time (min)
Particle density	40	300	7.5	1200	25
Water absorption	40	700	15	1150	20
Bloating ratio	20	300	7.5	1200	25
Loss on ignition	20	500	7.5	1200	20

**Table 16:** Particle density and water absorption of various lightweight aggregates.

Parameter	Experiment Number of Produced LWA				Commercial LWA	
	F4	F11	F15	F16	Norwegian Leca™ (0–4 mm)	Liapor™ 8 (4–8 mm)
Particle density (g/cm^3^)	1.18	1.27	0.89	0.89	1.26	1.47
Water absorption (%)	1.9	1.4	1.4	3.0	10.4	11.5

**Table 17:** Test results for high performance LWAs produced by the commercial tunnel kiln.

Particle Density (g/cm^3^)	Water Absorption (%)	Dry Loose Bulk Density (kg/m^3^)	Crushing Strength (MPa)
1.56	1.4	819	12.4
